# Periostitis

**Published:** 1887-09

**Authors:** Wm. Jefferson Guernsey

**Affiliations:** Philadelphia


### PERIOSTITIS—PULSATILLA.

Wm. Jefferson Guernsey, M. D., Philadelphia.

M. G., æt. thirty-eight years, married. On returning from a shopping expedition in the fall of 1883 she found her left heel quite sore, which she supposed to have been due to a nail in the shoe. In a short time it seemed to improve by rest, but it soon became very tender on walking, and as the trouble grew unbearable she called in her physician, a prominent allopath, who pronounced it periostitis. The soreness was so great as to compel her to use two crutches, the least pressure upon it being intensely painful. The doctor finally gave the case up as curable only by scarification of the bone. This she was unwilling to consent to without other advice, and a second physician was called who indorsed the diagnosis, and finding his efforts to relieve as fruitless agreed with his confrère that nothing but scarification would save the bone. A third and a fourth were summoned with a like result, each telling her that the operation was her only salvation. All this time (about two years) she could not move, even about the house, without both crutches, and seldom ventured more than a few doors from home with them. Finally, as a last resort before consenting to the butchery proposed, she yielded to the suggestion of a relative, whom I attended, and concluded to try Homœopathy, much against the wishes of her husband and friends, who were clamoring for a more heroic procedure. I had little doubt of curing the case and told her so, commencing treatment at once. She was a woman of good physique and without a complaint, save this, which made her a hermit indeed. I saw her first on the 13th of October, 1885, and treated her without effect for seven months. Why she continued so long under my unsuccessful treatment, against the combined protestations of all who knew her, I cannot imagine, unless she saw in my sanguine manner that I knew she *could* be cured and meant to do it. During this unfortunate period she was beset by constant inquiries from her old physicians, one of whom called and stated that he had come as a personal *friend* to urge upon her the extreme danger of delay, and another provoking incident occurred in her next-door neighbor asking a mongrel homœopathist what he thought of the treatment, and his replying that it was all nonsense, as there was "nothing in Homœopathy to reach such cases." I had given her Hyperic., Ruta, Arnica, Sil., Cycla., and Caust. in the order named, the latter only doing any good.

On the 19th of May, 1886, I took a careful review of her case, eliciting the following history: She never had been ill until her eighteenth year, when she had had pneumonia, from which she rallied slowly. Six years after that her first child was born, labor being instrumental and the child still-born. From this she was ill a long time. Her second and only other child was born two years after, from which she was much debilitated and terribly despondent for a long time. I judged from many little hints and from a knowledge of the physicians who had treated her that she had received more Quinine than food, and upon inquiry learned that she had taken enormous quantities of the drug when her heel trouble was first under treatment, and that the doctors had kept her on a liquid diet for three weeks, which meagre fare had doubtless intensified the virulence of the medicine. She was of a gentle temperament, inclined to be lachrymose without much cause, and while the *Materia Medica* did not seem to call for Pulsatilla, I found many general symptoms in Bœnninghausen.

The following were in bold type:

*Remedies affecting the heel:* Caust., Graph., Ig., Led., Na.-cb., Puls., Sab., Sep.

*Sole of the foot:* Cup., Mur. ac., Phos. ac., Puls., Sul., Tar.

*Bones of lower limbs:* Merc., Phos., Puls., Ruta, Sil., Staph.

*Bones inflamed:* Merc., Puls., Sil., Staph.

In small-caps the following:

*Periosteum inflamed:* Puls., and others.

*Painfulness of bones:* Puls., and others.

*Painfulness of periosteum:* Puls., and others.

*Sensibility of bones:* Puls., and others.

Pain, as if ulcerated in bones: Puls., and others.

Besides this, Puls. is a left-sided remedy. I gave her the CM (Fincke) every three hours for three days. Since that date it has been smooth sailing. She has had at odd intervals, as she seemed a little worse, Puls.$^{5c}$ (Tafel), Puls.$^{m}$ (Tafel), Puls.$^{52m}$ (Fincke), Puls.$^{cm}$ (Fincke), Puls.$^{cm}$ (Johnstone), and Puls.$^{mm}$ (Fincke), but always Pulsatilla.

As improvement set in I requested her to use but one crutch, then two canes, then one cane, and now for six months she has not carried anything of the kind, walks miles, and has not, save after excessive use, the slightest tenderness, which will quickly disappear after a dose or two of Puls. No local treatment whatever was allowed nor even rubbing used.

Last fall, on being sent for, I found her crying, and she told me that she was pregnant. Her menses were now three weeks overdue, and she was feeling some nausea at times. Amid a flood of tears she stated that she had sent for me to produce an abortion, as she knew another confinement would kill her. If I would not do it *she* would. I talked medicine, theology, and every other point to no avail. So, rising to go, I said : " I cannot prevent you from doing this thing, but as your physician I have one right—will you, on your honor, promise that if you do do it you will tell me of it." She replied that she would. "Very well," said I, "I will call to-morrow, and if you inform me that you have not changed your mind I shall never enter your house again." The next day I found her crying again, but this time the tears were from righteous humiliation and repentance, and she gave me the solemn promise I wanted.

Examining her case, I found distress in the epigastrium and hypogastrium, flying neuralgic pains and dyspnœa from a warm room. Telling her she had no positive indications of pregnancy anyhow, I left *Pulsatilla*, which brought on a healthy colored menstruation in two days and relieved all her other symptoms. She has had slight attacks of indigestion at times—always indicating and always cured promptly by Puls. And I think that unless this medicine cheats grim death forever out of this piece of humanity, I shall see to it that there is planted upon her grave a little wind-flower.

